# “I *know* all the things I should be doing …”: accounting for mental health and illness in an online mental health discussion forum during the COVID-19 pandemic

**DOI:** 10.1186/s40359-023-01424-8

**Published:** 2023-11-06

**Authors:** Grace Horwood, Martha Augoustinos, Clemence Due

**Affiliations:** School of Psychology, University of Adelaide, Adelaide, SA 5005 Australia

**Keywords:** Mental health, Mental Illness, Discourse analysis, Discursive psychology, Online discussion forums, Stigma

## Abstract

**Background:**

Mental health is highly correlated with a person’s social and economic circumstances, and the recent COVID-19 pandemic made this connection uniquely visible. Yet a discourse of personal responsibility for mental health often dominates in mental health promotion campaigns, media coverage and lay understandings, contributing to the stigmatisation of mental ill-health.

**Methods:**

In this study, we analysed how the concept of ‘mental health’ was discursively constructed in an online mental health peer-support forum in Australia during 2020, the period of the first two waves of the COVID-19 pandemic. An approach informed by Critical Discursive Psychology was employed to analyse all posts made to a discussion thread entitled “Coping during the coronavirus outbreak” in 2020, a total of 1,687 posts.

**Results:**

Two main interpretative repertoires concerning mental health were identified. Under the first repertoire, mental health was understood as resulting largely from the regular performance of a suite of self-care behaviours. Under the second repertoire, mental health was understood as resulting largely from external circumstances outside of the individual’s control. The existence of two different repertoires of mental health created an ideological dilemma which posters negotiated when reporting mental ill-health. A recurring pattern of accounting for mental ill-health was noted in which posters employed a three-part concessive structure to concede Repertoire 1 amid assertions of Repertoire 2; and used disclaimers, justifications, and excuses to avoid negative typification of their identity as ignorant or irresponsible.

**Conclusions:**

Mental ill-health was commonly oriented to by forum posters as an accountable or morally untoward state, indicating the societal pervasiveness of a discourse of personal responsibility for mental health. Such discourses are likely to contribute to the stigmatisation of those suffering from mental ill-health. There is a need therefore for future communications about mental health to be framed in a way that increases awareness of social determinants, as well as for policy responses to effect material change to social determinants of mental health.

## Background

Mental health and illness have been the subject of concern in Australia for some time, and the recent COVID-19 pandemic only increased such concerns [1]. While elevated levels of mental distress among the population receded once lockdowns ended, it is likely that post-pandemic levels of distress remain slightly above pre-pandemic levels [2]. Given the overall ineffectiveness to date of efforts in Australia to reduce rates of mental distress, mental illness and suicide [3, 4] there is a need for further critical reflection upon current approaches to this problem.

A large body of recent and historical research has demonstrated that social, economic and structural factors such as loneliness and social isolation, unemployment or precarious employment, low educational attainment, poor housing and living conditions and inequality are strongly correlated with poorer mental health [5, 6]. In both mental and broader public health policy in Australia, resources have primarily been directed towards treatment and behaviour change at an individual level, rather than on addressing upstream factors such as social determinants [7]. During the COVID-19 pandemic the impact of external factors upon mental health was made uniquely visible, and the post-pandemic phase is thus an opportune time to progress the public conversation to increase awareness of the social determinants of mental health.

### Mental health stigma

The World Health Organization (WHO) states that mental health is “a state of mental well-being that enables people to cope with the stresses of life, realize their abilities, learn well and work well, and contribute to their community” [8]. For the purposes of this article and following Zayts-Spence, Edmonds [9], we use the term ‘mental health’ to refer to a positive state of well-being as per the WHO definition, ‘mental illness’ to refer to a clinically diagnosable disorder, and ‘mental distress’ or ‘mental ill-health’ to refer to any other mental state associated with significant distress or impairment in functioning.

Stigmatising attitudes towards those with mental illness are common in Australia [10] and elsewhere. How mental health and illness are understood, and in particular what is seen as the cause of mental illness, is likely to have important implications for stigma [11]. Two major forms of mental illness stigma are perceptions of dangerousness, and attributions of personal responsibility [12]. ‘Personal responsibility’ stigma tends to be highest in relation to less severe conditions such as depression and anxiety [13], likely because these are seen as more controllable than conditions such as schizophrenia. Acceptance of public stigma and application to oneself can translate into self-stigma, which can lead to lowered self-esteem [14] and reluctance to seek help [15].

The widespread promotion of a bio-medical model of mental illness has corresponded with a reduction in personal responsibility stigma, however, stigma has not reduced overall; likely because biological explanations contribute to a perception that individuals with a mental illness are inherently and permanently defective [16]. In contrast, a social determinants of health [17] approach considers individual-level factors within the context of multiple layers of social, socio-economic, structural, cultural and environmental factors influencing health and health behaviours. Given that there is a tendency for people to under-estimate the impact of social factors on health [18], another means of breaking down mental health stigma could be to increase awareness of social determinants of mental health.

One of the key ways in which the status quo in health is reinforced and maintained is through discourse and language [19]. Mental health and illness are construed as such through discourse, with certain discourses becoming dominant in particular historical and cultural contexts [20]. Prevailing societal discourses and cultural norms will to some extent shape how states of mental health and illness are understood and experienced [21]. At the same time, discourses of mental health and illness may be challenged or shaped in new ways by individuals in interaction [20]. It is therefore important as part of mental health promotion and stigma reduction efforts to consider larger societal discourses as well as prevalent lay understandings.

### Online mental health discussion forums

During 2021–2022, nearly 5% of Australians reported accessing mental health resources using digital technologies, rising to 14.3% of those with a 12-month mental disorder [22]. Online discussion forums are one of the major resources accessed by those seeking help or information concerning mental health online [23]. Online forums have benefits to users of cost, anonymity, ability to self-pace, ease of access, round-the-clock availability, and opportunity to meet others with similar experiences [23, 24]. Previous qualitative research in the Australian context, conducted by researchers from a range of disciplines including public health, media studies, linguistics and health sciences, has indicated that users of online mental health forums are often socially or geographically isolated; and use forums to access social connections, as well as the expertise of peer mentors with lived experience, as an alternative to professional help [25–28]. Given the impact of the COVID-19 pandemic on mental health, and the reduced availability of in-person support during lockdowns, it is likely that the use of online discussion forums was high during this time.

Online discussion forums are ideal sites for discursive research into how people construct meaning, objects and identities, and negotiate issues such as authenticity and accountability, in a naturalistic social setting [29]. The use of naturalistic data foregrounds the concerns and thoughts about health that are relevant to participants as they go about their everyday lives, rather than those of the researcher [30]. Previous discursive or conversation analytic research analysing data from online discussion forums has done so in relation to a range of mental illness-related issues including psychiatric diagnosis [31], mental illness identities [32], bipolar disorder [33], depression [34, 35], post-natal depression [36], and anxiety [37]. However, we have not been able to locate any discursive studies which have examined how the more positive concept of ‘mental health’ is constructed in mental health discussion forums, and there are few qualitative studies analysing mental health forum data during the COVID-19 pandemic. One such study was carried out by Zayts-Spence, Tse [38], who used ethnographic and thematic methods to analyse mental health-related threads in an online support group for mothers in Hong Kong during the pandemic, finding that posts typically took the form of ‘troubles-talk’, especially in the face of potentially conflicting societal ideals of ‘good mother’ and ‘good worker’.

In this study, we examine posts made to an Australian mental health peer-support forum during the COVID-19 pandemic, analysing how the concept of ‘mental health’ was discursively constructed by forum posters, and in particular, how attributions of responsibility for mental health were made (i.e., whether mental health and ill-health were primarily attributed to individual factors, or to external factors such as social determinants) and associated consequences in terms of stigma.

### Data

The data for this study was sourced from the website of a major Australian mental health organisation, which focuses on the common conditions of depression and anxiety. Among other resources, the organisation hosts online peer-support forums on a range of mental-health-related topics. As a reputable, government-funded, non-profit mental health organisation, this organisation’s forums afford users a strong foundation of institutional trust and established norms and values. The forums are publicly and freely available to anyone to view, though (free) registration as a member is required to post, and membership is restricted to Australian residents. Forums are monitored and facilitated by two or three paid moderators and a larger number of unpaid but trained peer mentors. All forums are group discussions (there are no private conversations); and members can post text-based messages of up to 2,500 characters to any discussion thread at any time, or reply specifically to the posts of others using the ‘reply’ button on each post.

The forums, though publicly available to view, also afford users anonymity. The website’s Member Terms, which users must consent to upon registration, state that the website is a public place, and recommend that posters use a pseudonymous username and do not post personal information that they would not be comfortable sharing with a stranger or that might enable them to be identified. Username and forum role (e.g., moderator, community member) is the only information which is displayed publicly. Members are not permitted to share personal contact details, such as location, phone number or email address, with other members. All posts are reviewed by a moderator prior to being published online, but once published remain visible online permanently.

On 13 March 2020, soon after the COVID-19 pandemic was declared, a new forum thread entitled “Coping during the coronavirus outbreak” was created by a moderator. The data set for this study is comprised of all posts made to this thread between 1 and 2020 and 31 December 2020, a total of 1,687 posts. We focused on 2020 as this was the period of the first two waves of the pandemic in Australia, and the year during which most discussion activity occurred. Posts were downloaded by the first author on 13 September 2022.

Key ethical concerns in this study were the potentially sensitive and personal nature of posts; the likelihood that posters may include a vulnerable population, namely those with a mental illness; whether the discussion thread constituted a public or a private space (and how participants were likely to have viewed it); and the potential impact of this research upon posters, for example when posts are reproduced outside of their original domain [29, 39]. Central to addressing these concerns were the issues of informed consent and protection of privacy. Though all forum members have consented for their posts to be made publicly available online, as Stommel and Rijk [40] note, users of online platforms often still regard online interactions as private in some sense, and often do not consider consent to website terms and conditions as a sufficient substitute for consent to use of their data by researchers.

In this study, obtaining informed consent from forum users was impracticable as posts were anonymous, identities and contact details of posters were not known nor available to us as researchers, and researchers are not permitted to post on the forums about their research. Anticipated benefits of this research included the potential to reveal important and unique insights into lay understandings of mental health through the analysis of naturalistic talk and interaction which took place at the height of the pandemic. Following consideration of the above ethical concerns and of guidelines by the Association of Internet Researchers [41], we deemed posts to the online forum from which our data was sourced to be in the public domain already, meaning that informed consent from participants was not required. However, we endeavoured to minimise risks to participants by not naming the website from which the data was sourced and by not including poster usernames or dates of posts with published extracts. Ethics approval for this study was granted by the School of Psychology Human Research Ethics Sub-Committee at the University of Adelaide, approval number 22/92.

## Methods

Analysis in this study drew upon the methodological framework of Discursive Psychology (DP), which studies psychological issues and objects as they are constituted in interaction through the use of discourse and language [42]. In particular, we draw upon the ‘synthetic’ approach to DP [43] known as Critical Discursive Psychology (“CDP”), in which the analysis of how talk is put together at a micro level is combined with consideration of the wider ideological context in which talk occurs. Key analytic concepts in CDP include interpretative repertoires, subject positions and ideological dilemmas [44]. Interpretative repertoires have been described as “coherent ways of speaking that form over time, becoming easily recognisable arguments, assumptions, metaphors, figures of speech, and images” [45]. Subject positions are the different standpoints or identities made available to speakers in interaction [46]. Ideological dilemmas are the tensions or conflicts inherent in a culture’s ‘lived’ ideologies, which tend to be dilemmatic in nature [44]. Accounts are typically organised rhetorically by speakers so as to counteract an alternate construction of events or objects [47].

In this study, analysis was carried out in several stages [48]. Firstly, the first author read and re-read the data set repeatedly to build familiarity, noticing and recording patterns of language, recurring themes, and commonly invoked categories, particularly how posters constructed, understood and oriented to the concept of ‘mental health’. Secondly, recurrent discursive constructions or ways of talking about the topic of mental health were identified and grouped together, particularly with regard to what posters were ‘doing’ or accomplishing. Third, building upon the first two steps and successive re-readings of the data, the main interpretative repertoires concerning mental health were identified. Fourth, consideration was given to the subject positions or identities made available by these interpretative repertoires, as well as whether any ideological dilemmas were evident in the data. Fifth, illustrative extracts from the data that exemplified each repertoire were purposively selected by the first author and analysed at the micro level to show how linguistic and rhetorical devices were commonly used to construct mental health in certain ways. Finally, the interpretative repertoires, subject positions and ideological dilemmas noted to be prevalent in the data were considered in light of the wider social, cultural and ideological context, including what speakers might have been doing in that context by invoking or resisting certain repertoires and subject positions.

## Results

In the discussion thread from which our data set was drawn, entitled “Coping during the Coronavirus outbreak”, the opening post by a moderator invited members to respond to the question, *“What are you doing to look after your wellbeing during this time?”* Three days later, there was another post by the moderator containing more specific questions to guide contributions, as follows:

What thoughts are helpful for you at the moment?

What are you doing to remain connected?

How are you taking care of yourself and others?

What are you finding helpful in reducing your anxiety during this time?

As such, discussion on this thread would have been significantly shaped by this guidance.

In the data, ‘mental health’ was mainly conceptualised by posters in a functional sense as being able to cope with the demands of daily life. The term ‘coping’ was primarily used to refer to emotion-focused coping, that is, regulating emotional responses to a stressor through behavioural and cognitive strategies [49]. Overall, mental health and coping were constructed using two main interpretative repertoires, which are outlined below along with representative extracts. These two repertoires explained mental health and coping in different ways, which produced an ideological dilemma. Repertoires 1 and 2 are outlined to provide the background for this dilemma, before the ways in which posters negotiated the resultant dilemma and accounted for states of poor mental health are analysed in more depth. Extracts from the data have been presented with the original style of the authors preserved, but line numbers have been added for ease of reference in our analysis.

### Repertoire 1: Mental Health/coping as the result of individual behaviours of ‘self-care’

A prevalent interpretative repertoire was that mental health can be maintained or improved through the individual regularly practising multiple mental health-promoting, ‘self-care’ behaviours. Mental health was typically constructed in this way using multi-part lists and causal attributions, as exemplified in Extract 1:

#### Extract 1

1. I have certain things I do every day because they keep me mentally healthy. They support
2. my sense of self-esteem, personal identity and agency. They are: water my plants,
3. squeeze and drink a citrus fruit, some random stretching, my French lesson, look at trees
4. and feel awe for nature, write a gratitude list, exchange affirmations with three friends via
5. text, and connect properly, emotionally, with at least three different other people.
6. I enjoy all that stuff so it’s no burden. It’s effective for me… Bang for my buck.
7. Things I do **nearly** daily for my mental health are:
8. walk outside, even a short walk just for circulation and fresh air.
9. volunteering, giving it away, service to others, whatever you want to call it.
10. meditate with an app.
11. aromatherapy.
12. journal (a speech recording or in writing, whatever I feel like).
13. As you can see, it’s all about self-care, gentleness, recharging, connection and joy.
14. Most important, do what works for YOU.

Extract 1 was part of a post made by a frequent forum user, in response to a post by a first-time forum user who reported experiencing anxiety, depression and low motivation during lockdown and asked whether anyone had “any ideas about creating structure”. In Extract 1, two lists are included of things the poster does to maintain their mental health - the first (lines 2–5) listing eight things that the poster reports doing every day (line 1), and the second (lines 8–12) listing five things that the poster reports doing nearly every day (line 7). Previous conversation analytic research has found that three-part lists are normative in interaction [50], reflecting a preference for brevity and functioning to construct a description as summary, complete or adequately representative of a class of items. Lists longer than three parts (“*long lists*”) have received less research attention, possibly because of a focus by researchers on spoken interactions, which have certain constraints including a bias towards single turn completion units, a preference for progressivity and a default to minimisation [51], making the use of long lists less likely. In contrast, computer mediated settings involving written interactions such as online discussion forums are likely to offer different affordances and have different constraints [52]. In this case, the peer-support forum from which data was sourced has a 2,500-word limit for posts, and posts are published online permanently as a resource for the public to access, affording users more opportunity and reason for the use of long lists.

In the data set generally, multi-item lists of self-care practices such as these were frequently shared by forum members. One of the key reasons why users utilise connections with others via online mental health peer-support discussion forums is to share and receive information and practical advice [26]. The sharing of lists of self-help or recovery strategies has previously been found to be a common way in which more experienced mental health forum members share their expertise for the benefit of newer members, with posts containing such lists often revisited and used as a reference point over time [25].

Long lists may have a distinct rhetorical function in interaction: namely they construct disparate items as members of the same category, and infuse these items with a subjective meaning or evaluation that supports an overall argument [53]. In Extract 1, various activities of daily life that might be seen as ordinary are grouped together in long lists with more recognisably therapeutic activities, the effect being that all these activities are categorised as therapeutic techniques carried out towards the goal of maintaining mental health. While the items on such lists might be customised to some extent (line 14), the assumption in the data generally was that everyone would have such a list of their own.

In Extract 1, the poster makes an explicit causal link between performance of self-care behaviours daily and the outcome of mental health (line 1, line 7), a *script formulation* [54] that was common in the data set. This causal sequence was typically constructed in the data as routine, recognisable or expected; implying that deviations from this sequence would be a breach of usual expectations, and thus potentially accountable events [54]. As illustrated in Extract 1, self-care behaviours were usually described as simple actions within the individual’s ability to implement (line 6). Therefore, according to Repertoire 1, mental health was constructed as being largely (though not necessarily entirely) within the control of the individual.

Even when the limits of self-care for mental health were tested by the extraordinary circumstances of the pandemic, Repertoire 1 was often re-asserted. This is illustrated in Extract 2 - part of a post made shortly after the announcement of a second lockdown in one Australian city:

#### Extract 2

1. In terms of actually managing ongoing anxiety around the situation, I’d say it’s time to
2. amplify self-care routines. Find your inner child. Do something each day that makes you
3. excited. I’m not talking about something you just ‘look forward too.‘ I mean to do
4. something that makes your heart skip a beat and makes you smile without realising.
5. Build a pillow fort. Do it- this one was fun for me. Get your kids involved if you have any.
6. Do a backyard experiment. Make that dream purchase (online if you can!). Sing karaoke.
7. Offer to foster a shelter puppy if it’s within your means. Do something you won’t get
8. bored of or won’t regret committing too anytime soon (insert all of my neglected home
9. improvement projects if you are anything like me lol).
10. I hope this helps and is something a little different to the usual “distract yourself whilst
11. staying at home” activities we usually see.

This post was made in response to a specific question from another member, “I wonder can anyone think of anything that has helped them?”, but also referenced posts from several other members expressing feelings of frustration, before offering the above advice. In Extract 2, the anxiety caused by the external situation is acknowledged (line 1), however a discourse of self-care is not only reinforced, but intensified to match the intensification of external pressures (lines 1–2). As in Extract 1, Extract 2 includes a long list of suggested self-care behaviours (lines 5–7), also to be practised daily (line 2) to manage anxiety (line 1), and causal links are made between these behaviours and increases in positive emotions (lines 2–4). Thus, the prevalent script formulation that the regular performance of a range of self-care behaviours will lead to the individual maintaining mental health, or managing mental ill-health, is reinforced.

### Repertoire 2: Mental health/coping as the result of external circumstances.

Repertoire 2 represented an alternative explanation for mental health. According to this repertoire, mental health is heavily and unavoidably impacted by circumstances and pressures external to the individual, and therefore is often not within the individual’s control. Under Repertoire 2, self-care routines were constructed as having limited efficacy for mental health while social, economic, and structural determinants of mental ill-health remained unresolved, as exemplified in Extract 3:

#### Extract 3

1. I think this pandemic is preventing me from finding any sort of happiness at the moment.
2. I’ve been unemployed since before the first lockdown, went through a million job
3. interviews in-between with no leads, I’ve been heartbroken (maybe twice), I went
4. through health issues (but recovered after 1.5 months of hell) and I realise I have no
5. friends that actually care about me. I would have travelled the world for the first time in
6. my life and would have given me space to feel hopeful again but I can’t even do that. I
7. have taken up knitting which is nice but it doesn’t let me escape from the sheer boredom
8. and reality that I have no one.

The post which Extract 3 was from did not appear to be a response to any other post specifically, however the post immediately prior to this one included a suggestion that “by keeping our minds active and our body’s safe, we can prevail through these uncertain times”. Extract 3 begins, like Extract 1, with an explicit attribution of causality, but in this case the poster’s unhappiness is attributed to factors external to the individual and outside of their control, namely the pandemic (line 1). Interestingly, another long list [53] is included, consisting of five external factors that have impacted on the poster during the pandemic (lines 2–6). Some of these problems appear to have occurred due to the pandemic, and some appear to be problems that pre-dated the pandemic or would have occurred regardless. Nevertheless, these five elements are grouped together in a list, building shared meaning in the form of support for the poster’s contention that they are being prevented from finding any happiness (line 1). In lines 6–7 the poster makes a concession [55] to an alternative repertoire of self-care reminiscent of Repertoire 1 – “I have taken up knitting” (lines 6–7), but this is followed with a reassertion of Repertoire 2 (lines 7–8), in that behaviours such as knitting do not provide an escape from broader personal and structural challenges. That is, mental health is dependent not only upon individual lifestyle and self-care factors, but also upon social, structural, and economic determinants.

Extract 4 is another example of a poster drawing upon Repertoire 2:

#### Extract 4

1. I have thought of calling the help line, but I can’t imagine what anyone there could say to
2. help. I know all the things to do, reading here and in the media - getting a routine going,
3. eating and sleeping well, exercising, reading a book, craft, talking with friends, avoiding
4. social media and the news as much as possible etc. etc., but none of that replaces my
5. big need for face to face conversations with laughter and smiles, a hug, things to look
6. forward to, not worrying about our future, the Australian economy.

This post from which this extract was taken was the third in a sequence of three posts. In the opening post, this forum member expressed difficulties in coping due to loneliness and isolation. A forum moderator replied, expressing empathy, and providing details of online resources and a telephone helpline staffed by mental health professionals. The above extract is from the third post in the exchange, in which the forum member replied to the moderator. In lines 1–2, the poster appears to anticipate that, were they to call a mental health help line, they would most likely be provided with advice about self-care along the lines of Repertoire 1, and therefore sees little point in calling. In lines 2–4, the poster demonstrates that their knowledge of self-care behaviours is already adequate, citing a long list of seven self-care behaviours, followed by “etc etc” (line 4). Generalised list completers such as “etc” signal not only that there are more list items that could be named, but that there are many more such items [50]. As such, standard lists of self-care behaviours are constructed as lengthy, involved, possibly endless, and, by implication, of limited effectiveness.

In lines 5–6, the poster then cites another long list of external factors, most being conditions arising from the pandemic, that are currently impacting their mental health. These are described as “big need(s)” (line 5), constructing them as inherent human needs, and not matters of choice. The use of two lists set in contrast - as indicated by the contrastive conjunction ‘but’ (line 4) - serves to construct the two lists as different categories of things. The poster states that the items on the first list, which are all individual lifestyle factors, cannot replace (line 4) the need for a solution to the problems on the second list, and implies that their mental health or ill-health is primarily dependent on the items on the second list. The items on the second list correspond to social determinants of mental health which are larger than individual lifestyle factors, such as social support networks, and socio-economic conditions [6]. Thus, mental health is constructed as being mainly the result of circumstances external to the individual and outside of their control.

### Ideological dilemma: accounting for states of mental ill-health/not coping

The existence of two different explanatory repertoires of mental health created an ideological dilemma that posters were obliged to negotiate, especially when it came to explaining a current state of mental ill-health. As discussed above, under Repertoire 1 there was a prevalent script formulation that performance of a standard suite of behaviours of self-care would prevent mental ill-health, and an assumption that practising such behaviours was within the ability of every individual. Experiencing a state of mental ill-health was thus commonly oriented to by forum posters as an accountable matter. A pattern that occurred repeatedly was that, when reporting a current state of mental ill-health, posters without solicitation presented an account to others for their mental ill-health.

An *account* is a statement offered by a social actor to explain behaviour that is regarded in the cultural context as odd, wrong or untoward [56]. Social relations are necessarily accountable phenomena [57], and members of moral communities work continuously to preserve social order through holding each other to account relative to normative rules or standards [58]. Descriptions and accounts of behaviour are always accomplishing ‘moral work’ of some kind [57], and are occasioned, constructed and received based on the orientation of speakers to certain sets of assumptions or explanations available in the immediate interactional and wider cultural context [59]. Scott and Lyman [56] identified two main types of account: *justifications*, in which responsibility for an act is accepted but the wrongness of the act is denied; and *excuses*, in which the wrongness of an act is accepted but responsibility for it is denied. Hewitt and Stokes [60] added the concept of *disclaimers*, which are accounts offered prospectively for upcoming conduct that might be problematic, as opposed to the usual retrospective nature of accounting.

In the data, posters appeared to anticipate that a state of mental ill-health would be interpreted by others as evidence of a failure to perform standard routines of self-care, and thus accounts centred around the question of whether they had been performing self-care behaviours, and if not, why not. Accounts for mental ill-health were noted to take a remarkably similar form. First, posters would report a current state of mental ill-health or not coping, often with references to external pressures (i.e., Repertoire 2). Second, posters would state that they knew about the self-care behaviours that can be practised to maintain mental health (i.e., Repertoire 1), usually beginning with the words, “I know …”. Third, usually prefaced by the contrastive conjunction “but”, posters would re-assert continuing mental ill-health, often with further references to external pressures (Repertoire 2). This structure is similar to a structure Antaki and Wetherell [55] term a *show concession*. Concessive patterns in general are recognised as “the negative or contradictory counterpart of causal constructions” [61], that is, they occur against the backdrop of an assumed causal relationship, and describe a situation which differs from the expected sequence of events. Show concessions characteristically consist of a three-part structure of assertion, concession and re-assertion, and hinge around two markers - a marker of concession such as “okay”, “I agree”, “obviously”, etc., and a reprise marker signalling contrast to the concession, most commonly “but” [55]. The main rhetorical function of show concessions is not to concede but to undermine a competing position by displaying awareness and understanding of it before challenging it.

A show concession structure is evident in Extracts 3 and 4 above, which have been used to illustrate Repertoire 2. The accounts in Extracts 3 and 4 might be said to take the form of *justifications*, in that any failure to perform standard self-care behaviours is constructed as permissible, given their ineffectiveness in solving the real problem of difficult circumstances. More usual in the data, however, was that posters did not directly dispute Repertoire 1, but instead struck a more delicate balance by embedding an affirmation of Repertoire 1 within descriptions of experiences inconsistent with the causal assumptions of Repertoire 1. Extract 5 is an example of this:

#### Extract 5

1. Since Covid-19 rolled in, a deep seated anxiety has surfaced that I think I always knew
2. was present. I would say I’ve been a bit of a minor worrier all my life. These days I feel
3. constantly anxious, and the uncertainty of what the future holds or looks like I am finding
4. paralysing. My emotions roller coaster from anger, to fear, to total numbness and I am
5. crying a lot.
6. The second lockdown hit me hard and I did speak to a psych who told me my feelings
7. were all natural in these unprecented times. I *know* all the things I should be doing,
8. establishing a routine, eating well, sleeping well, exercising, reaching out to friends. I am
9. doing most of these - although I have little energy or motivation to exercise - I have taken
10. up yoga and pilates, which can help sometimes with ‘staying present’.
11. In the last week my role at work was made redundant due to my then employer being in
12. crisis management mode, at the same time as Melbourne has gone into hard lockdown. I
13. am despondent and so incredibly sad.

The author of this post identified themselves as a first-time poster who had recently joined the forums. Their post did not appear to be made in reply to any previous post. In Extract 5, a three-part structure is evident. In lines 1 to 7, the poster describes a current state of distress, along with references to the external factors of Covid-19 (line 1), uncertainty about the future (line 3) and lockdowns (line 6) – a description consistent with Repertoire 2. In lines 7 to 10, the poster inserts a reference to Repertoire 1, stating that they know about, and are carrying out, routines of self-care. Then in lines 11 to 13, the poster continues describing their state of mental distress (lines 12–13) along with further references to external pressures of unemployment (line 11) and lockdowns (line 12).

Despite repeatedly linking their decline in mental health to external circumstances (line 1, line 6) and social determinants such as unemployment (lines 11–12), the poster orients to their mental distress as an accountable matter, using moral language such as “should” (line 7) in reference to preventive routines of self-care. The poster appears to anticipate that, in response to a report of mental ill-health, others will question whether they know about, and practice, routines of self-care. The middle section of this three-part structure (lines 7–10) thus arguably functions as a *disclaimer* - a pre-account used to ward off in advance anticipated negative evaluations of future conduct [60].

The poster’s marked emphasis on their knowledge of routines of self-care (line 7) defends against a potential undesired typification of their identity as someone who is ill-informed or ignorant. As Stivers, Mondada [62] argue, the epistemic domain is morally ordered, and people hold one another accountable for their normative rights and responsibilities in respect of knowledge. The stylistic choice “I *know*” pre-emptively and emphatically establishes the speaker’s epistemic status as equal to that of other forum members (implying that a lack of knowledge would be a moral failing). The speaker’s epistemic access is reinforced by their report, positioned immediately prior in line 6, of a recent consultation with an expert in the form of a psychologist.

In line 8, the poster also pre-emptively wards off any typification of their identity as irresponsible, citing a long list of five standard items of self-care (line 8) and stating that they are doing most of these (line 9) and more (line 10). In this way, the poster signals agreement with and commitment to Repertoire 1, despite current experiences of mental ill-health. Regarding the effectiveness of the self-care strategies being practised, the poster adds a limited, hedged and vague statement that two of the items listed, yoga and pilates, “can help sometimes” (line 10). Although the poster’s report of continued mental ill-health in lines 11–13 would appear to call into question the commonly assumed causal relationship between self-care routines and mental health, the poster does not address this dissonance directly.

A similar three-part structure occurred repeatedly in the data set when posters reported and accounted for current states of mental ill-health. In cases where (unlike the poster in Extract 5) a poster disclosed that they were *not* performing daily routines of self-care behaviours, an *excuse* was also typically included as part of their account. Excuses are “socially approved vocabularies for mitigating or relieving responsibility” for an acknowledged moral transgression [56]. Some of the most common forms of excuse are a claim to an impairment of knowledge (“I did not know”) or an impairment of ability (“I was not able”). In the data for this study, the excuse of a lack of knowledge almost never occurred, whilst the excuse of impaired ability was the norm. Extract 6 is an example:

#### Extract 6

1. Just signed up to this community because I just need support. I struggle pretty badly with
2. anxiety normally so this pandemic has just pushed me overboard.
3. I’m taking all the precautions against it but all I can think about is running out of food. I
4. live in a small country town and people from the cities have been coming to buy from our
5. shops, leaving them just as empty but with longer delivery times. All I can think about is
6. running out of food and my children going hungry.
7. Sometimes I can bring it all back into perspective but other times I’m just locked into
8. panicked thoughts.
9. I think I mainly just wanted to vent that, I know that I need to get some self care
10. measures in place but I’m just struggling.

The author of this post identified themselves as a new forum member (line 1) and did not appear to be responding to any specific previous poster, beginning this post with “Hello all”. In Extract 6, a similar three-part structure occurs to that which occurred in Extract 5. Lines 1–8 begin by describing a current state of mental ill-health in the form of anxiety (line 2) and panicked thoughts (line 8), along with references to the external factors of the pandemic (line 2, line 5). In lines 9–10, the poster references Repertoire 1, saying, “I know that I need to get some self care measures in place”, thereby disclaiming an identity as ignorant, and affirming self-care routines as normal, expected and appropriate behaviour. In line 10, this is followed by the conjunction “but” and a re-assertion of mental ill-health. Unlike the poster in Extract 5, in Extract 6 the poster implies that they do not currently have a regime of self-care behaviours in place (lines 9–10). The last phrase, “I’m just struggling”, thus functions both as a re-assertion of mental ill-health, and as an excuse for not having self-care measures in place. Previous conversation analytic research has shown that when an action or response is dis-preferred, accounts and excuses “overwhelmingly cluster around the issue of ability” [63], the advantage of this kind of account being its ‘no blame’ quality - unlike accounts that might indicate a lack of thought, effort, interest or desire. Throughout Extract 6, the poster’s thoughts and mental health are constructed as outside of their control at present (lines 2, 3, 5, 10) due mainly to external pressures (line 2), thereby mitigating their responsibility; though the poster acknowledges and agrees that self-care by individuals is important.

## Discussion

In this study, we examined how mental health was constructed in an online mental health discussion forum in Australia in 2020, during the first wave of the COVID-19 pandemic. It was found that mental health was constructed using two main interpretative repertoires. Repertoire 1 constructed mental health as being largely the result of individuals performing a suite of self-care behaviours daily. Many posters reported that routines of self-care were effective in helping them to cope during the pandemic, and the effectiveness of self-care for mental health has been supported by subsequent research [64]. Repertoire 2 constructed mental health as largely the result of social and economic factors external to the individual – a perception also borne out by research during the pandemic showing a correlation between mental health and various socio-demographic factors [65]. Both Repertoires 1 and 2 were prevalent in the data, creating an ideological dilemma which posters sought to negotiate, especially when reporting a current state of mental ill-health. Seemingly because of the causal assumptions associated with Repertoire 1 – that responsible performance of self-care behaviours would prevent mental ill-health - posters repeatedly oriented to mental ill-health as an untoward state and pre-emptively offered accounts in the form of justifications, disclaimers and/or excuses.

In discursive psychological research on health topics, the key issue is not what people say *about* health but what people *do* when talking about health [66]. The offering of an account is a sign that issues of morality, identity and personal worth are at stake. Tileaga [59] notes that accounts in naturally occurring interaction are a valuable subject of inquiry because they reveal what is regarded in a particular cultural context as wrong, untoward, or problematic. According to Billig, Condor [47], talk about health and illness is always ideological, because ‘health’ describes the ideal subject according to the prevailing ideology of a society or culture. Talk about health and illness is also always dilemmatic, in that the ill person must show that they are a ‘special case’, i.e., unwell enough to be unable to carry out usually expected duties; but also that they are normal or ‘fit for society’, i.e., compliant with social norms and expectations, in order to be seen as worthy of help [67]. When people account for health and illness, therefore, they do not simply express beliefs or attitudes, but “construct their state of health as part of their ongoing identity in relation to others … making claims about themselves as worthy individuals, as more or less ‘fit’ participants in the activities of the social world” [67].

That forum posters experiencing mental ill-health oriented to this as a state requiring an account, even amidst the exceptional circumstances of the pandemic, indicates that mental ill-health is still widely viewed as morally problematic. This is perhaps an unintended consequence of mental health public awareness campaigns that are perceived as placing the burden of responsibility on individuals to manage their mental health [68]. In the typical patterns of pre-emptive accounting for mental ill-health observed, what posters appeared to be doing was signalling affirmation of socially approved standards of behaviour – that is, agreeing with the importance of carrying out regular routines of self-care (Repertoire 1) - while at the same time reducing attributions of individual moral responsibility for their mental ill-health by citing external pressures – attributions which posters appeared to anticipate would otherwise be made. In this way, posters were able to retain an identity as informed and responsible citizens, i.e., fit for society, despite currently experiencing mental ill-health.

At a macro level, the patterns observed in this study indicate the societal pervasiveness of notions of individual responsibility, self-control and lifestyle characteristic of the ‘new public health’ [69]. In modern neoliberal societies, the ideal citizen is one who assumes full responsibility for their own happiness, health, and productivity, and rationally maximises these [70]. Within ideological tendencies of healthism and individual responsibility, “self-care” is one of the main discourses by which responsibility for health and illness is transferred to the individual [71]. While self-care and self-help might in many cases be effective and even empowering, there is always a risk that this language will foster the illusion that individual responsibility is sufficient and there are no constraints on people’s choices, leading to poor health being seen as the result of individual moral failings [71]. Such societal discourses and norms are likely to contribute to mental illness stigma and self-stigma [72]. The results of this study indicate that there is a continuing need to re-frame the public narrative towards seeing mental ill-health as related to social determinants and other macro-level factors, rather than the focus being mainly on individual-level factors.

Limitations of this study include the lack of demographic information for forum posters, the possibility of over-representation of certain groups such as people with a mental illness, and difficulties in accurately discerning the intended meaning of posts due to the lack of opportunity to ask speakers for clarification or more detail. In addition, this study analyses posts made in 2020 during the first two waves of the COVID-19 pandemic in Australia, to a peer-support forum specifically dedicated to coping during the pandemic, and findings thus may not be generalisable to other contexts.

## Conclusion

The results of this study suggest that the dominance of Repertoire 1 in societal discourses of mental health in Australia has some unproductive effects in terms of personal responsibility stigma. To reduce stigma, it is recommended that, in future mental health communications, any advice which may increase attributions of personal responsibility, such as self-care advice, be tempered with an acknowledgement of the limits of self-care in the face of difficult circumstances and larger social and structural determinants of mental health. In addition, language and metaphors can be chosen that construct mental ill-health as a societal-level and not just an individual-level problem. For example, a metaphor for unequal circumstances which several posters used was that of boats in a storm - as one said: “We are not all in the same boat, we are in the same storm. Some of us are in yachts, and some of us are in rowboats, some of us are clinging to driftwood. We are all in different boats in the same storm”.

More fundamentally, there is a need for policy responses aimed at reducing the social determinants impacting negatively on mental health, targeted at multiple levels [73]. As outlined by Fisher [7], social policy in relation to mental health and wellbeing should address factors including access to meaningful and socially valued work, societal and living conditions that promote social connectedness, and local communities that engage members as active participants. As indicated by the findings of this study, it is unlikely that significant progress in reducing population rates of mental distress and ill-health will occur without changes to the larger social conditions and environments in which individuals live and work.

## Data Availability

The dataset analysed during the current study is available in the Figshare repository, DOI: 10.25909/23429675.
